# The clinical application of cancer immunotherapy based on naturally circulating dendritic cells

**DOI:** 10.1186/s40425-019-0580-6

**Published:** 2019-04-18

**Authors:** Kalijn F. Bol, Gerty Schreibelt, Katrin Rabold, Stefanie K. Wculek, Julia Katharina Schwarze, Andrzej Dzionek, Alvaro Teijeira, Lana E. Kandalaft, Pedro Romero, George Coukos, Bart Neyns, David Sancho, Ignacio Melero, I. Jolanda M. de Vries

**Affiliations:** 1 Department of Tumor Immunology, Radboud Institute for Molecular Life Sciences, Nijmegen, the Netherlands; 2 Department of Medical Oncology, Radboud university medical centre, Nijmegen, the Netherlands; 3 Radiotherapy & OncoImmunology Laboratory, Radboud university medical centre, Nijmegen, the Netherlands; 4 Immunobiology Laboratory, Centro Nacional de Investigaciones Cardiovasculares `Carlos III`, Madrid, Spain; 5 Department of Medical Oncology, Universitair Ziekenhuis Brussel, Brussels, Belgium; 6 Miltenyi Biotec GmbH, Bergisch-Gladbach, Germany; 7 Center for Applied Medical Research, University of Navarra, Pamplona, Spain; 8 Department of Oncology, Lausanne University Hospital, Lausanne, Switzerland; 9 CIBERONC, Madrid, Spain

**Keywords:** Dendritic cells, Natural dendritic cells, Plasmacytoid dendritic cells, Myeloid dendritic cells, Conventional dendritic cells, Cross-presenting dendritic cells, Cancer, Immunotherapy, Vaccination

## Abstract

Dendritic cells (DCs) can initiate and direct adaptive immune responses. This ability is exploitable in DC vaccination strategies, in which DCs are educated ex vivo to present tumor antigens and are administered into the patient with the aim to induce a tumor-specific immune response. DC vaccination remains a promising approach with the potential to further improve cancer immunotherapy with little or no evidence of treatment-limiting toxicity. However, evidence for objective clinical antitumor activity of DC vaccination is currently limited, hampering the clinical implementation. One possible explanation for this is that the most commonly used monocyte-derived DCs may not be the best source for DC-based immunotherapy. The novel approach to use naturally circulating DCs may be an attractive alternative. In contrast to monocyte-derived DCs, naturally circulating DCs are relatively scarce but do not require extensive culture periods. Thereby, their functional capabilities are preserved, the reproducibility of clinical applications is increased, and the cells are not dysfunctional before injection. In human blood, at least three DC subsets can be distinguished, plasmacytoid DCs, CD141^+^ and CD1c^+^ myeloid/conventional DCs, each with distinct functional characteristics. In completed clinical trials, either CD1c^+^ myeloid DCs or plasmacytoid DCs were administered and showed encouraging immunological and clinical outcomes. Currently, also the combination of CD1c^+^ myeloid and plasmacytoid DCs as well as the intratumoral use of CD1c^+^ myeloid DCs is under investigation in the clinic. Isolation and culture strategies for CD141^+^ myeloid DCs are being developed. Here, we summarize and discuss recent clinical developments and future prospects of natural DC-based immunotherapy.

## Background

In 1973 Cohn and Steinman discovered a new type of immune cell, the dendritic cell (DC) [1], which plays an important role in the induction of specific immunity. DCs are sentinels of the immune system, as they are deployed throughout the body and monitor their surroundings for antigens and danger signals derived from pathogens or tissue damage. They are the most potent antigen-presenting cells, able to initiate and modulate specific immune responses.

In their immature state, DCs mainly reside in lymphoid and peripheral tissues where they recognize and capture antigens. Upon receiving an activating stimulus in the presence of inflammatory signals, DCs undergo maturation and migrate to lymphoid organs. DC maturation is associated with functional and morphological changes, an essential process for T-cell activation. The immature phenotype of DCs is mainly characterized by a low surface expression of MHC I and II molecules and co-stimulatory molecules and a high capacity for phagocytosis that mediates sampling of antigens [2]. DCs activated by so-called “danger signals” become highly motile, their endocytic and phagocytic receptors are down-modulated, and chemokine receptors that foster migration to lymphoid organs are upregulated. Furthermore, cell surface expression of MHC molecules and adhesion/co-stimulatory molecules, such as CD40, CD54, CD80, CD83, and CD86 is upregulated, and production of specific cytokines is induced [3]. In the lymphoid organs, mature DCs present processed exogenous peptides to naive CD4^+^ T-cells via MHC class II and endogenous peptides to CD8^+^ T-cells via MHC class I. In addition, some DCs have a superior capacity to cross-present exogenous antigens on MHC class I to CD8^+^ T-cells [2], which is important for the induction of cytotoxic T-cell responses against tumor cells. Effective T-cell priming in the lymphoid tissues requires three signals between DCs and T-cells: antigen presentation via the MHC-peptide complex (signal 1), stimulation via co-stimulatory molecules from the DC to the T-cell (signal 2), and immune-stimulatory cytokines in the microenvironment (signal 3) [3].

The ability of DCs to initiate and direct adaptive immune responses is exploited for cancer immunotherapy, especially in DC vaccination. With DC vaccination, mature DCs loaded with tumor antigens ex vivo are injected into cancer patients to induce tumor-specific effector T-cells that aim to recognize and eliminate cancer cells and induce immunological memory to control tumor growth [4]. In the majority of clinical DC vaccination trials conducted so far, DCs differentiated ex vivo from monocytes or CD34^+^ progenitors have been used, since naturally circulating DCs (nDCs) are present in the blood but only constitute about 1% of blood mononuclear cells. However, through the development of efficient isolation techniques, the use of nDCs has recently become feasible. In this review, we summarize and discuss recent clinical developments of DC-based immunotherapy with nDC subsets, comprising completed and ongoing clinical trials.

### Lessons from DC vaccination with moDCs

Prompted by excellent results against transplanted mouse tumors with bone marrow-derived DC cultures, the first DC vaccination trials were performed in the late nineties. The effect of various DC vaccination parameters on immunological and clinical outcome of vaccination has been studied in numerous small phase I/II clinical trials in cancer patients. Most of these studies have been performed with monocyte-derived DCs (moDCs), due to their easy differentiation protocol in vitro.

#### Maturation of moDCs

MoDCs are mostly HLA-DR^+^/MHC-II^+^ CD11c^+^ BDCA3^−^ and frequently express CD16, CD14 and DC-SIGN, due to their monocytic origin [5]. Their functions and appearance are very divers, likely due to the inflammatory context they are differentiating in and the variety of cytokine cocktails that are used for their activation ex vivo. From the first clinical studies it became evident that proper activation of the DCs is of major importance for DC vaccination of cancer patients, otherwise antigen-specific tolerance is induced rather than antitumor immunity [6–8]. Besides inducing expression of molecules important for T-cell activation, maturation of DCs leads to upregulation of chemokine receptors which promotes the migration of injected DCs to the lymph nodes and is thus of importance for vaccination efficacy [9].

In vivo, DC maturation is triggered by pathogens or tissue injury. In vitro, this can be mimicked by incubation with pathogen recognition receptor agonists or a cocktail of proinflammatory cytokines. A cytokine cocktail consisting of tumor necrosis factor (TNF)α, interleukin (IL)-1β, IL-6 and prostaglandin E2 (PGE2), or monocyte-conditioned medium with TNFα and PGE2 are the most widely used methods for moDC maturation [10, 11]. Whether this is the best cocktail to induce maturation remains controversial since PGE2 may confer immunosuppressive effects [12, 13]. To further induce DC activation, mimicking viral infection, type I interferons have been added to the cocktail [14]. More recently, the use of Toll-like receptor (TLR) ligands [15, 16] or electroporation with mRNA-encoding proteins that induce DC maturation [17] has been explored. The latter methods yield DCs that produce higher levels of IL-12, which favors the differentiation of T helper 1 (Th1) cells and promotes activation of potent CD8^+^ effector T-cells.

#### Antigen loading and administration of moDCs

To induce a tumor-specific immune response in cancer patients, DCs should be loaded with relevant tumor antigens. The most widely used techniques for antigen loading of DCs vaccines are pulsing DCs with MHC-binding peptides of tumor-associated antigens (TAA), corresponding long peptides or proteins, TAA-encoding mRNA, or tumor lysate. All antigen-loading techniques have their advantages and disadvantages; none has proven to be superior to the others thus far, however, loading with both MHC class I and class II epitopes seems beneficial for the quality of the induced immune response [18].

For DC vaccination, it is crucial that DCs migrate to the T-cell areas of the lymph nodes after administration. In murine models it was shown that intravenously injected DCs mostly accumulate in highly vascularized organs like spleen, lungs, kidneys and liver, rather than lymph nodes and fail to induce skin-homing T-cells [19, 20]. Migration studies with labeled DCs demonstrated that after intradermal injection, only 2–4% of the injected cells migrate to draining lymph nodes, whereas most of the injected cells die at the dermal injection site and were cleared by macrophages [21–23]. After intranodal injection, injected cells accumulate in the injected node and subsequent draining lymph nodes [23, 24]. By leaving the DCs directly at the site of interaction with T-cells, this route of administration obviates the need for DCs to migrate. However, cells need to be injected under ultrasound guidance.

Comparison of induced immune responses after DC vaccination via different routes of administration showed variable results [23, 25, 26]. Intradermal injection seems to yield superior T-cell responses in terms of tumor recognition and cytokine production [23], which might in part be explained by the fact that, after intradermal migration, only the most mature and most potent DCs reach the lymph nodes, in contrast to intranodal injection, where also nonviable and less mature DCs are directly delivered into the lymph nodes.

#### moDCs in clinical trials: the outcome

Thus far, numerous phase I/II clinical trials with moDC vaccines have been performed in cancer patients. Side-effects were minimal and included grade 1–2 flu-like symptoms, fever and local injection site reactions. Grade 3–4 toxicity is very uncommon after DC vaccination but can occur with more potent moDC formulations [15, 27–29]. Thus, DC vaccination can be concluded to be safe when used as monotherapy.

Although safe and able to induce anticancer immunity, so far objective clinical responses have only been achieved in a minority of patients after moDC vaccination, usually around 5–15% of metastatic cancer patients. However, despite the lack of clear benefit in objective clinical responses, a trend to survival benefit was reported in most studies [28]. This is often seen with immunotherapy, as it takes time until the full potential of the anti-tumor response is reached and sometimes delayed objective clinical responses occur, or only stable disease is achieved that nevertheless can be highly durable. This dissociation between objective response and overall survival (OS) is hampering the clinical implementation of DC vaccination as larger randomized clinical trials would be required when survival rather than tumor response is used as a primary endpoint. Furthermore, most trials were conducted with widespread metastatic patients in which tumor-induced immune suppression is probably too strong to overcome with DC vaccination alone. Still, numerous small trials improved the quality of the DC vaccines over the years and moDC vaccination still holds promise for clinical application. Combination of DCs with other forms of anticancer treatment might be a solution to overcome tumor-induced immune suppression. For example, the combination of moDCs with anti-CTLA4 blockade in advanced melanoma patients showed an encouraging response rate of 38%, with all complete responders (*n* = 7) still free from progression and off-therapy more than 5 years after the initiation of DC therapy [30]. Another option to obtain more robust antitumor responses, might be adjuvant DC vaccination, when only minimal tumor load in present [31, 32]. Data from phase III clinical trials are needed to substantiate the results of the successful smaller trials. Recently, OS data of a randomized phase III clinical trial in glioblastoma patients treated with a moDC vaccine in combination with chemotherapy and radiotherapy were published [33]. The median OS seems promising compared to literature, but due to the cross-over trial design groups within the trial cannot be properly compared and progression-free survival (PFS) data is awaited.

## Naturally circulating dendritic cells

MoDCs may not be the best DC source for immunotherapy, since they have been described to have decreased migratory capacities towards the site of T-cell interaction by exhaustion of the cells [34], probably due to the artificial differentiation by cytokines and extensive ex vivo culture periods. nDCs may be a potent alternative for moDCs, as the brief ex vivo exposure of nDCs might preserve the functional capabilities of the cells and prevent exhaustion. Although direct comparison of nDCs and moDCs in clinical trials have not been performed (yet) to validate the in vitro data. In addition, The Cancer Genome Atlas reveals that specific nDC subsets, rather than moDCs, are associated with improved survival in diverse cancer types [35–37]. Although the isolation of monocytes from the blood has a much higher yield, direct isolation of nDCs is now feasible and facilitates robust standardization for use in multicenter trials and, eventually, standard care.

**Fig. 1:**
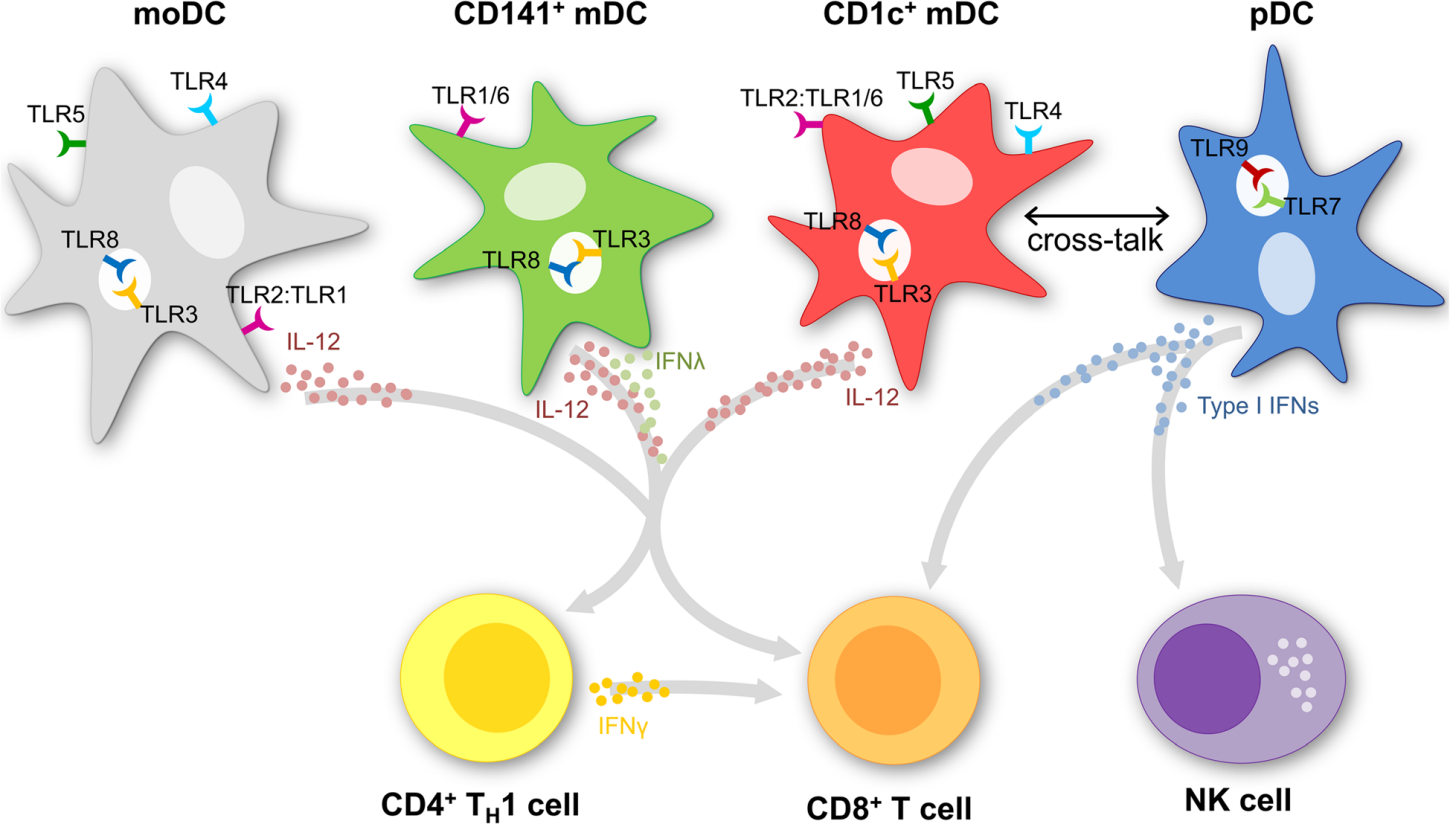
Dendritic cell subsets. Dendritic cells can be differentiated from monocytes (moDC), which are often used in clinical trials because of their high yield. The naturally circulating dendritic cells can now also be enriched by immunomagnetic isolation. The naturally circulating dendritic cells can further be divided in myeloid (CD141^+^ and CD1c^+^ mDC) and plasmacytoid dendritic cells (pDC). The subsets differ in function, localization, phenotype and cytokine production

nDCs comprise a heterogeneous population of cells. Functional, transcriptomic and proteomic reports identified the major circulating DC subsets, which are distinguished by distinct surface markers [38–40]. Human DCs can be subdivided in two main subsets (Fig. 1); plasmacytoid DCs (pDCs) and myeloid/conventional DCs (mDCs). These subsets differ in function, localization, and phenotype [41]. pDCs have a plasma cell-like shape and are specialized in viral antigen recognition, they largely lack expression of extracellular TLRs and are the main producers of type I interferons (IFNs) [42, 43]. They are mainly localized in T-cell areas of lymph nodes and express BDCA2 and BDCA4 [41, 44]. pDCs appear to be predominantly tolerogenic in the context of cancer and correlate with poor prognosis [44]. However, when properly activated, they have the ability of cross-presentation and may therefore be potent inducers of antitumor responses [45–47]. Activation of pDCs induces upregulation of MHC molecules and costimulatory molecules, allowing efficient priming of CD4^+^ and CD8^+^ T-cells. The secretion of large amounts of type I IFNs can induce Th1 polarization as well as activation of innate immune cells, such as macrophages and natural killer cells [45, 48–50]. Type I IFNs produced by pDCs are also beneficial for antigen cross-presentation by mDCs [51]. mDCs are mainly localized in the marginal zone of lymph nodes and express MHC II and CD11c [48, 52, 53]. They express extracellular TLRs (TLR1, TLR2, TLR4–6) and endosomal TLRs (TLR3 and TLR8), which are responsible for the ability of mDCs to secrete the Th1 skewing cytokine IL-12 upon activation [43]. The mDC population can be further subdivided into two classes based on surface expression into CD1c(BDCA1)^+^ DCs (cDC2s) and CD141(BDCA3)^+^ DCs (cDC1s), with the CD1c^+^ mDCs being the most potent T-cell stimulators of these subpopulations [43, 54, 55]. CD1c^+^ mDCs also consist of two subsets, both populations stimulate T-cell proliferation but differ in their potential for cytokine secretion [56]. CD1c^+^ mDCs seem specialized in immunity against bacteria and fungi, whereas CD141^+^ mDCs are specialized in detection and uptake of necrotic cell debris of virally infected cells or tumor cells and cross-presentation of derived antigens to CD8^+^ T-cells [57–59]. Especially a subset of BDCA3^+^XCR1^+^CLEC9A^+^ cells seems to be a superior cross-priming DC subset in humans [57–61]. They can migrate from peripheral organs to lymph nodes and efficiently cross-present cell-associated antigens to induce CD8^+^ T-cells [39, 40, 60, 62, 63]. Most recently, myeloid DC have also found to be of pivotal importance in “relicensing” the antitumor activity of cytotoxic T-cells within the tumor microenvironment [35, 37]. Furthermore, a subset of CD16^+^ ‘non-classical’ monocytes with DC-like characteristics is found in human blood [64].

In animal models, it was shown that mature antigen-loaded pDCs or mDCs can prime tumor-specific T-cell responses that result in tumor eradication [65–67]. In a murine glioma model, vaccination with mDCs was superior to pDCs in survival benefit [67]. Recent studies suggest that pDCs and mDCs cooperate and act synergistically. In mice, pDCs were shown to induce tumor-specific CD8^+^ T-cell responses and enhance the ability of mDCs to present tumor antigens to T-cells [68, 69]. In human, mDCs and pDCs were shown to activate each other after specific stimulation of one of the subsets with TLR ligands in vitro [53]. Combining the two subsets in one vaccine might thus exploit their functions simultaneously and increase their immunotherapeutic potential [70].

### Isolation of nDCs for clinical use

**Fig. 2:**
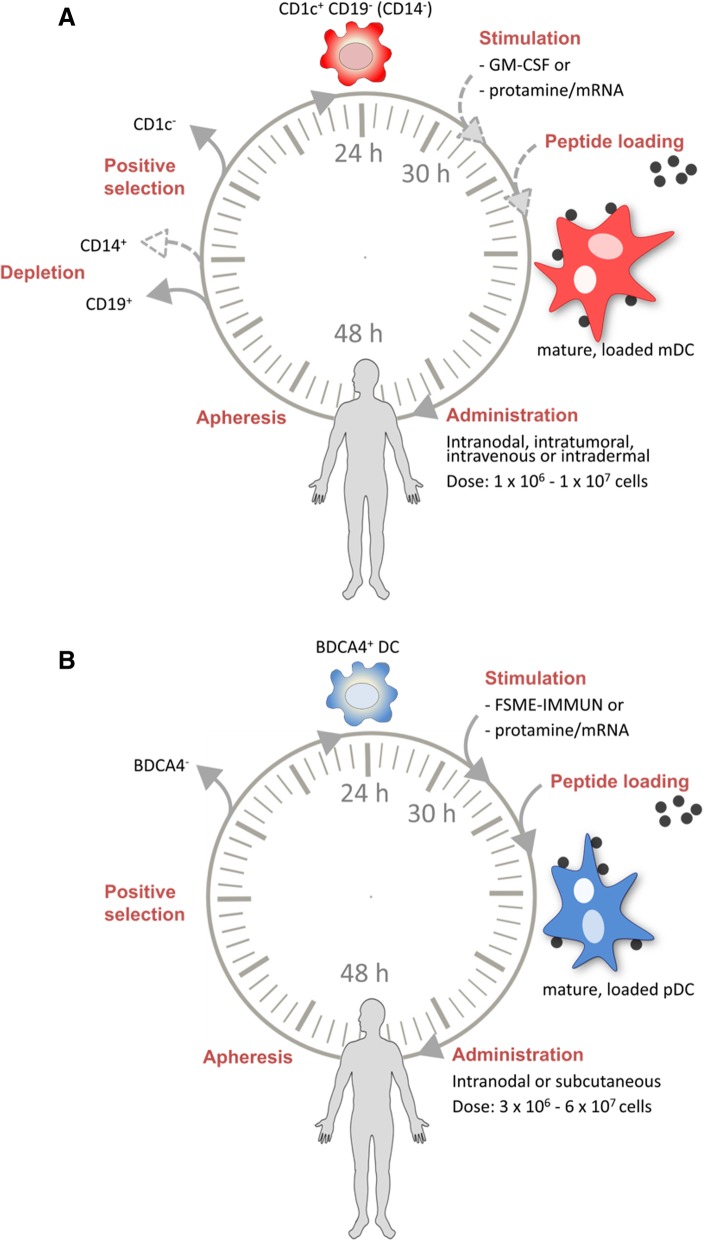
Production protocols for naturally circulating dendritic cells. Schematic overview of the (**a**) CD1c^+^ myeloid dendritic cell (mDC) and (**b**) plasmacytoid dendritic cell (pDC) production protocols and vaccination strategy of the various clinical trials

Production of DC vaccines is a labor-intensive process comprising numerous open handling steps such as density gradient cell processing, cell washing steps, cell labeling/separation, cell culture, final product formulation and cryopreservation. The complexity of the process makes it prone to failure and requires experienced personnel and complex protocol development under GMP guidelines. Therefore, manufacturing of clinical grade DC vaccines can be performed only in highly specialized institutions and existing manufacturing processes are hardly transferable, which in turn limits patient’s access to this kind of therapy. To overcome these limitations, there is a growing effort in the field to develop standardized, robust and reproducible protocols for production of DC vaccines. In this regard, automation of such processes is a major step forward as it limits operator-dependent variance and thereby reduces deviations not only between individual production runs but also between productions that are performed at different clinical centers. Miltenyi’s CliniMACS Prodigy® platform consists of an integrated device, clinical grade buffers and reagents and a single-use tubing set that allows for temperature and atmosphere-controlled cell culture. It has been designed to automatically perform all cell handling steps in a closed system with minimal user interaction for highest reproducibility [71]. In addition, the closed system reduces the need for complex class A clean-room resources as it can be operated in a class C GMP-environment. The production of nDC vaccines consists of positive selection to enrich pDCs and/or mDCs using magnetic antibody-coupled beads, optionally preceded by depletion of monocytes and B cells (Fig. 2). Currently, two additional processes are under development aiming for the isolation of cross-presenting CD141^+^ mDC and panDC (pDC + CD1c^+^ mDC + CD141^+^ mDC). Addition of CD141^+^ mDCs may further improve nDC vaccines, since this mDC subtype is highly efficient in antigen cross-presentation and able to secrete IFNλ and IL-12 upon activation [57–59, 72].

### nDCs in clinical trials: the vaccines

Currently, 9 clinical trials with autologous nDC vaccination are performed in cancer patients, of which three are completed [73–75], one was terminated (ACTRN12607000450415), and 5 are ongoing (NCT02574377, NCT02692976, NCT02993315) of which 2 are still recruiting patients (NCT03707808, NCT03747744). The trials included patients with either melanoma, prostate cancer or any solid tumor. Three trials focus solely on CD1c^+^ mDC vaccination and one trial solely on pDC vaccination, whereas in the remaining trials that are being performed the combination of both nDC cell types is studied or CD1c^+^ mDC vaccination is combined with other immunotherapeutics (Table 1). All trials performed isolation of DCs using the immunomagnetic CliniMACS® isolation system. Another method to obtain DCs from an apheresis product is the enrichment of DCs by density centrifugation. This method was mainly performed before the emergence of magnetic separation. Magnetic separation is a faster, less labor-intensive method and results in a purer population of cells with a sufficient yield compared to density centrifugation. As density centrifugation does not result in pure populations, studies performing density gradient isolation were not included in this review. Among these are studies using the clinically approved sipuleucel-T for metastatic castration-resistant prostate cancer [76], which only contains a small fraction of CD54^+^ DCs, i.e. stimulated DCs, among T-cells, natural killer cells, monocytes, and B-cells. In essence, the vaccination product is the result of peripheral blood mononuclear cells (PBMCs) obtained from apheresis activated by a fusion protein between prostate acid phosphatase and granulocyte-macrophage colony-stimulating factor (GM-CSF) [77]. The contribution of the diverse cell types to the activity of the intravenously administered vaccination product remains unclear.

**Table 1:**
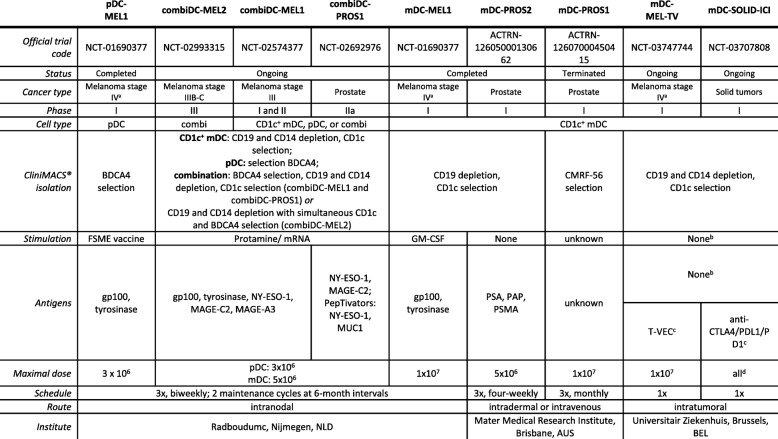
Clinical trials with natural DC vaccination

^a^Stage IV melanoma patients or irresectable stage III melanoma patients

^b^Anticipated capture of tumor antigens and maturation in vivo

^c^mDC vaccination in combination with T-VEC intratumorally or anti-CTLA4 and anti-PDL1 intratumorally and anti-PD1 intravenously

^d^All mDCs obtained from the leukapheresis are injected

Abbreviations: FSME, Frühsommer-meningoencephalitis; GM-CSF, granulocyte-macrophage colony-stimulating factor; mDC, myeloid dendritic cell; pDC, plasmacytoid dendritic cell; T-VEC, Talimogene laherparepvec. (source: clinicaltrials.gov and anzctr.org.au)

#### mDC vaccines

The first clinical trial with mDCs, in 2007, was conducted in hormone-refractory metastatic prostate cancer patients (mDC-PROS1). Isolation of mDCs was performed by positive selection for CMRF-56, an early DC activation/differentiation antigen expressed by mDCs after in vitro culture for 6 h [78, 79]. This single-step isolation procedure showed higher yields as well as less variable purity compared to the isolation by density gradients performed in the clinical setting until then, but the product still contained about 30% CD14^+^ and CD19^+^ cells [80]. Therefore, this study was terminated and as the study results are not published, no conclusions can be drawn concerning the isolation and culture method.

In subsequent trials, the CD1c^+^ mDCs were selected by depletion of B cells (CD19^+^) followed by positive selection of CD1c^+^ cells. In the trial with metastatic melanoma patients (mDC-MEL1), initiated in 2010, this procedure resulted in an average purity of 93% and a yield between 27 × 10^6^ and 96 × 10^6^. The isolated cells were stimulated by GM-CSF, resulting in semi-mature mDCs that are HLA-ABC/DQ/DR^+^ CD86^+^ and showed variable CD83 and CD80 expression [74]. A trial conducted with mDCs in metastatic prostate cancer patients (mDC-PROS2) used the same selection technique and obtained a similar purity (median 82%) and yield (28-101 × 10^6^). However, no stimulation was added in this trial and phenotyping established that all CD1c^+^ cells within the vaccines had a semi-mature phenotype (CD86^+^ CD40^−^ CD80^−^ CD83^−^) [75].

In 4 trials, to obtain CD1c^+^ mDCs, cells expressing the monocytic marker CD14 were also depleted in addition to CD19^+^ cells, since CD1c^+^ CD14^+^ cells were shown to suppress CD4^+^ T-cells and may severely hamper DC vaccine efficacy [81]. In two trials with completed patient accrual, stage III melanoma patients (combiDC-MEL1) or metastatic prostate cancer patients (combiDC-PROS1) were either vaccinated with mDCs, pDCs or the combination of both. In the two ongoing trials, the mDCs are neither fully matured nor loaded with antigen ex vivo, but injected intratumorally in combination with Talimogene Laherparepvec (T-VEC; mDC-MEL-TV), an oncolytic virus approved for non-visceral melanoma metastasis [82], or the immune checkpoint inhibitors anti-CTLA4 and anti-PDL1 intratumorally and anti-PD1 intravenously (mDC-SOLID-ICI). It is hypothesized that the semi-mature mDC capture tumor antigens and mature in vivo after intratumoral exposure to the co-injected T-VEC virus or immune checkpoint inhibitors, which have the potential to elicit antigen-dependent cellular cytotoxicity and complement-dependent cytotoxicity, thereby creating an inflamed tumor microenvironment.

To stimulate the DCs in the combination trials, DCs are activated with protamine/mRNA which can induce maturation of both pDCs and CD1c^+^ mDCs [47]. Although the two DC subsets do not express an overlapping repertoire of TLRs, single-stranded RNA is a suitable maturation stimulus as it is a ligand for TLR7 on pDCs and TLR8 on mDCs, inducing IFNα and IL-12 production, respectively. Furthermore, stimulation with protamine/mRNA was shown to result in an increase in the expression of MHC class I and CD86, and a variable expression of CD80 on both mDCs and pDCs. Consequently, the stimulated DCs were able to induce T-cell proliferation and activation [47]. This characteristic of protamine/mRNA to activate both CD1c^+^ mDCs and pDCs provides more flexibility in combining the two subsets in one vaccine. Preliminary data from these two trials shows a similar phenotype as in the preclinical study (unpublished data).

#### pDC vaccines

The pDCs, used in the combination trials and a trial with a single pDC vaccine in melanoma patients (pDC-MEL1), are isolated by BDCA4 bead-coupled antibodies, without any prior depletion step. Compared to mDCs, pDCs have a lower average purity of 75% and a yield between 13 × 10^6^ and 33 × 10^6^ cells [73]. Activation of the autologous pDCs is either performed with Frühsommer-meningoencephalitis (FSME) vaccine (pDC-MEL1) or protamine/mRNA (combination trials). In contrast to mDCs, GM-CSF is not suitable as stimulus for pDCs, as it does not efficiently activate pDCs to produce type I IFNs [74]. In contrast, FSME is a suitable maturation stimulus for pDCs as it triggers TLR7, leading to cytokine production and maturation [83].

Although outside the scope of this review, there is one trial with a pDC vaccine in melanoma patients (NCT01863108) using pDCs from an allogeneic cell line. This cell line is derived from a malignant leukemic pDC and cells are irradiated prior to administration to prevent further proliferation of pDCs in the patient [84]. The advantage of an allogeneic cell line lies in the abolishment of the limited cell yield and obviates the need for a leukapheresis. Although vaccination with allogeneic DCs will induce an allogeneic immune response, these responses may even be beneficial for the antitumor response [85]. Preclinical studies showed that using allogeneic DCs was safe and effective, however, the allogeneic DCs and the patient must share a HLA antigen, in this case HLA-A*02:01, to enable antigen presentation. Furthermore, there is a chance to develop immune reactions against the DCs itself. In the case of repetitive vaccination this would lead to unwanted killing of the allogeneic DCs by the immune system.

#### Administration and antigen loading

In all but two trials a cycle of three vaccinations with a 2 to 4-week interval was administered. In the 4 trials performed in Nijmegen, maximally two maintenance cycles were given in the absence of progressive disease. In the phase I trials combining mDCs with T-VEC or immune checkpoint inhibition intratumorally, currently only a single vaccination is given. Concerning the administration of the vaccine, the trials differ in the number of cells that are administered, the administration schedule, and the route of administration (Table 1). In all trials using antigen loading, peptide pulsing was performed. As electroporation procedures comes with moderate cell toxicity, this is a serious drawback for the scarce nDCs. The tumor antigens used differ between the trials, mainly due to the different tumor types expressing different antigens. The number of cells mainly depend on the yield of nDC isolation and ranges from 1 × 10^6^ to 1 × 10^7^ cells per vaccination. There is little evidence about the most effective cell number per vaccination, even for moDCs. In most trials intranodal injection was performed, again due to the scarcity of the cells.

### nDCs in clinical trials: the outcome

#### Monitoring immune responses

In all phase I/II trials, the primary and secondary endpoints were safety and immunological outcome. For immunomonitoring purposes, DCs were loaded with a control antigen in all trials performing antigen loading. Both trials with CD1c^+^ mDCs (mDC-MEL1, mDC-PROS2) used keyhole limpet hemocyanin (KLH) as a control antigen and as a source for T helper epitopes. In the mDC-PROS2 trial, KLH-specific antibodies could be detected by ELISA in the peripheral blood after vaccination in 4 of 12 patients, of which one patient already had KLH-specific antibodies prior to vaccination [75]. In the mDC-MEL1 trial, KLH-specific antibodies could be found in a similar percentage of patients (4 of 13 patients), with detectable KLH-specific antibodies prior to vaccination in 2 patients but with increased levels after vaccination. Also, T-cell proliferation upon stimulation with KLH was shown in 11 of 13 patients after the first round of vaccinations [74]. However, previously no correlation with survival and a strong KLH-specific T-cell response could be found in a cohort of 91 patients [86].

As pDCs do not have the capacity to take up soluble KLH [87], KLH cannot be used for immunomonitoring in patients vaccinated with pDCs. In the pDC-MEL1 trial, the FSME vaccine was used as a maturation stimulus and served as a control antigen. In this study, peripheral blood showed T-cell proliferation upon stimulation with FMSE in 10 of 14 patients tested, while FMSE-specific antibodies were present in 12 of 15 patients [73]. The data on immune responses against the control antigens indicate that nDC vaccination can effectively induce de novo immune responses in cancer patients. As different control antigens were used, no direct comparison of the efficacy of the induction of the novo immune responses between mDCs and pDCs can be made.

All published trials also analyzed the presence of tumor antigen-specific T-cells, either by FACS or ELIspot assays. In the mDC-MEL1 trial, tumor antigen-specific T-cells were detected in PBMCs of 4 of 12 patients [74]. In the mDC-PROS2 trial no tumor antigen-specific T-cells could be detected [88]. In the pDC-MEL1 trial, tetramer stainings were negative, however, after in vitro restimulation with antigenic peptides, an increase in tumor antigen-specific T-cells after vaccination could be detected in 7 of 15 patients [73]. This in vitro restimulation was not performed in the other trials.

Furthermore, delayed-type hypersensitivity (DTH) skin tests were performed in all 3 trials after (each round of) 3 vaccinations. In the mDC-PROS2 trial, DTH skin test were conducted with prostate-specific peptides. No skin reactions, pain/itching or erythema was observed to KLH or prostate-specific peptide. However, skin reactions were present against a control peptide (FMP) in 4 patients [75]. Unfortunately, no skin biopsies were taken for further analyses, although swelling/erythema of a DTH skin test does not correlate with the presence of tumor antigen-specific T-cells [89] but the presence of tumor-specific T-cells correlates with clinical outcome [86, 89]. Therefore, in both trials performed in Nijmegen biopsies were taken irrespective of induration of the DTH injection sites. Tumor-specific CD8^+^ T-cells were detected with tetramer stainings in 4 of 13 patients (mDC-MEL1) and 2 of 15 patients tested (pDC-MEL1) [73, 74]. Despite the small number of patients in the mDC-MEL1 trial, the correlation between the presence of tumor-specific T-cells and survival could be observed [74].

#### Toxicity and survival

Toxicity data for nDC vaccination thus far is limited, but the toxicity seems to be similar or even more favorable than with moDC (Table 2). To date, little can be concluded on the clinical efficacy of nDC vaccination. In both trials with metastatic melanoma patients, PFS was short in most patients. However, in the trial with CD1c^+^ mDCs, PFS was longer in patients with tumor-specific T-cells compared to patients without tumor-specific T-cells. Furthermore, despite the short PFS in most patients, OS seems to be relatively long. The median OS was 13 (mDC-MEL1) and 22 months (pDC-MEL1) [73, 74]. However, OS might be biased by subsequent treatments. In the mDC-PROS2 trial, the asymptomatic hormone-refractory metastatic prostate cancer patients showed a median OS of 18 months, including one patient alive over 5 years after enrollment [75]. Of the more recent trials, highly preliminary data of the intratumoral mDC vaccination in combination with immune checkpoint inhibitors (mDC-SOLID-ICI) showed a durable partial response (> 8 months) in a melanoma patient who previously progressed on immune checkpoint inhibition [90]. More robust survival data should be obtained from the first phase III trial with nDCs (combiDC-MEL2). This trial started in 2016 and studies vaccination with the combination of pDCs and CD1c^+^ mDCs compared to placebo in the adjuvant setting in stage III melanoma patients. Due to the recent approval of anti-PD1 antibodies and combined BRAF/MEK inhibition as adjuvant treatment, the inclusion had to be stopped before completing planned accrual. However, the circa 150 enrolled patients will provide valuable results in the near future.

**Table 2:** Baseline characteristics and outcome measures after natural dendritic cell vaccination

		pDC-MEL1	mDC-MEL1	mDC-PROS2
Cancer type		Melanoma	Melanoma	Prostate
Patients	Male/female	10/5	10/4	12/0
Age, years	Median (range)	52 (35–69)	50 (31–73)	69.5 (52–78)
Disease stage	M1a	1	4^a^	2
	M1b	8	2	5
	M1c	6	9	3
	Unknown	0	0	2
Line of systemic treatment	1st line	14	14	0
	2nd line or later	1	0	12^b^
Number of vaccines received	2 or 3	12	9	12
	6	2	2	–
	9	1	3	–
Vaccine-specific toxicity	Grade 1	6	4	n.a.
	Grade 2	0	1	n.a.
Immunological responses	Control antigen^c^ (blood)	T-cell: 10/14 Ab: 12/15	T-cell. 11/13 Ab: 4/13	T-cell: n.t. Ab: 4/12
	Control antigen (DTH)	n.t.	n.t.	4/12^d^
	Tumor antigen (blood)	7/15^e^	4/12	0/12
	Tumor antigen (DTH)	2/15	4/13	0/12^d^
Progression-free survival	Median (range; months)	4.0 (< 4–20)	2.8 (< 4–67+)	n.a.
Overall survival	Median (range; months)	22.3 (< 4–64)	13.3 (< 4–67+)	18 (6–40+)

^a^Including 1 irresectable stage III melanoma patient

^b^All patients received 2–4 lines of hormonal treatment. Four patients received prior chemotherapy

^c^T-cell proliferation upon stimulation with the control antigen (T-cell) and control antigen-specific antibodies (Ab) are shown

^d^Skin reaction tested only

^e^No tumor-specific T-cells were detected prior to restimulation

Abbreviations: n.a., not available; n.t., not tested

### Future perspectives: CD141^+^ mDCs, neoantigens and in vivo targeting of nDCs

Of the different nDC subsets, the CD141^+^ mDCs are the only subset that has not been explored in a clinical trial yet. The isolation of these cells is even more challenging because of their extreme scarcity in peripheral blood (0.2–0.3% of total mononuclear leukocytes). With recently developed isolation kits, the cells can be isolated with a purity of 70–85% after positive selection for CD141 and 3-6 × 10^6^ cells can be obtained from one leukapheresis. This subset is truly specialized in cross-presentation and a similar subset in mice, Batf3-dependent CD8α^+^ lymphoid or CD103^+^ DCs, were shown to be crucial for the induction of antitumor T-cell responses and tumor control [35, 37, 59]. These mouse equivalents of human CD141^+^ mDCs are also essential for recruitment of T-cells within the tumor and effective checkpoint antibody therapy [91, 92]. Moreover, there is a strong link of mDC infiltration in the tumor with increased survival in several cancers in The Cancer Genome Atlas [36, 37, 93]. Therefore, vaccination with this subset of CD141^+^ mDCs is postulated to result in superior antitumor immune responses in cancer patients and is being optimized for clinical application.

A different path to improve DC vaccines might the use of neoantigens. Neoantigens are generated by somatic mutations in the tumor. Exploiting neoantigens requires sequencing of the tumor of the patients and prediction of their MHC molecule binding capacity. Although labor-intensive and time-consuming, it is feasible and might be the future for antigen loading of DCs [94–96]. Alternatively, intratumoral injection of DCs is currently under evaluation for its potential to capture unknown neoantigens in vivo.

Another recent approach to exploit DCs for cancer immunotherapy is to target DCs subsets in vivo, by antibodies with activating agents and antigens [97]. Antigen bound to antibodies directed against surface receptors of DCs that are implicated in endocytosis, can lead to uptake of the antigen, loading on MHC, and subsequent induction of immune responses [98]. However, if these antibody-antigen conjugates are not accompanied by adjuvant to stimulate the immune system, tolerance rather than immunity might occur. The adjuvant can be given systemically, locally or specifically targeted to nDCs by antibody-coated (nano)particles loaded with both antigen and adjuvant [99]. The advantage of the latter approach is that adjuvants only activate those DCs that are targeted by the antibodies, thereby preventing systemic activation and toxicity, and conversely, that DCs loaded with antigens are also stimulated and matured with adjuvant, so no immature DCs are loaded with tumor antigens [100]. The main advantage of in vivo targeting strategies is the development of an off-the-shelf product. However, further research is needed before clinical trials can be started.

## Conclusion

Based on all the in vitro data, nDCs may be a potent and more practical alternative to moDCs. Currently, with immunomagnetic isolation the scarce nDCs can be obtained for DC vaccination. The advantage of nDCs lies in the rapid and highly standardized, automated production of the vaccines, which can improve the quality of the DC vaccines and enables multicenter trials. Furthermore, as nDCs are not artificially differentiated and only undergo a short ex vivo culture period, it is hypothesized that they retain their functional capabilities and avert exhaustion. The results from the few completed trials with nDCs show promising results with very limited toxicity. Subsequent trials as well as data from ongoing trials will have to substantiate the role of nDCs in DC-based immunotherapy as data is currently too limited to draw firm conclusions regarding nDCs and their comparison to moDCs. It will be interesting to investigate what DC vaccines can offer and whether their therapeutic effects can enhance those of checkpoint inhibitors when used in combination.

## Abbreviations

DC: Dendritic cell
DTH: Delayed-type hypersensitivity
FMSE: Frühsommer-meningoencephalitis
GM-CSF: Granulocyte-macrophage colony-stimulating factor
IFN: Interferon
IL: Interleukin
KLH: Keyhole limpet hemocyanin
mDC: Myeloid dendritic cell
MHC: Major histocompatibility complex
moDC: Monocyte-derived dendritic cell
nDC: Naturally circulating dendritic cell
OS: Overall survival
PBMC: Peripheral blood mononuclear cell
pDC: Plasmacytoid dendritic cell
PFS: Progression-free survival
PGE2: Prostaglandin E2
TAA: Tumor-associated antigen
Th1: T helper 1
TLR: Toll-like receptor
TNF: Tumor necrosis factor
T-VEC: Talimogene Laherparepvec
